# Characterization of miRNA and their target gene during chicken embryo skeletal muscle development

**DOI:** 10.18632/oncotarget.22457

**Published:** 2017-11-06

**Authors:** Endashaw Jebessa, Hongjia Ouyang, Bahareldin Ali Abdalla, Zhenhui Li, Auwalu Yusuf Abdullahi, Qingshen Liu, Qinghua Nie, Xiquan Zhang

**Affiliations:** ^1^ Department of Animal Genetics, Breeding and Reproduction, College of Animal Science, South China Agricultural University, Guangzhou 510642, Guangdong, China; ^2^ Guangdong Provincial Key Lab of Agro-Animal Genomics and Molecular Breeding and Key Lab of Chicken Genetics, Breeding and Reproduction, Ministry of Agriculture, Guangzhou 510642, China; ^3^ Department of Animal Nutrition and Feed Science, College of Animal Science, South China Agricultural University, Guangzhou 510642, Guangdong, China; ^4^ Department of Animal Production and Management, College of Animal Science, South China Agricultural University, Guangzhou 510642, Guangdong, China

**Keywords:** miRNA, chicken embryo, muscle

## Abstract

MicroRNAs (miRNAs) are non-coding RNAs that regulate mRNA expression by degradation or translational inhibition. We investigated the underlying molecular mechanisms of skeletal muscle development based on differentially expressed genes and miRNAs. We compared mRNA and miRNA from chicken skeletal muscle at embryonic day E11, E16 and one day post-hatch (P1). The interaction networks were constructed, according to target prediction results and integration analysis of up-regulated genes with down regulated miRNAs or down-regulated genes with up-regulated miRNAs with |log2fold change| ≥ 1.75, *P* < 0.005. The miRNA-mRNA integration analysis showed high number of mRNAs regulated by a few number of miRNAs. In the E11_VS_E16, comparison group we identified biological processes including muscle maintenance, myoblast proliferation and muscle thin filament formation. The E11_VS_P1 group comparison included negative regulation of axon extension, sarcomere organization, and cell redox homeostasis and kinase inhibitor activity. The E16_VS_P1 comparison group contained genes for the negative regulation of anti-apoptosis and axon extension as well as glomerular basement membrane development. Functional *in vitro* assays indicated that over expression of miR-222a and miR-126–5p in DF-1 cells significantly reduced the mRNA levels of the target genes CPEB3 and FGFR3, respectively. These integrated analyses provide several candidates for future studies concerning miRNAs-target function on regulation of embryonic muscle development and growth.

## INTRODUCTION

Skeletal muscle is the abundant tissue in the body and its main functions include supporting body structure, controlling motor movements and a major site of metabolic activity [1]. In chicken embryos, skeletal muscle growth occurs from the beginning of incubation through early post-hatch [2] and research using this system has made a significant impact on the fundamental biology of development [3]. This model system is ideal for genomic manipulations resources because it facilitates access to a developing embryo and RNA interference (RNAi) protocols can be easily adapted. In other way electroporation of chicken embryos and the use of RNAi to knock down gene expression are possible to make the chicken embryo a powerful model for the molecular study of development in vertebrate gene function [4]. MicroRNAs (miRNA) are non-coding RNAs that regulate diverse developmental and molecular processes by reducing gene expression at the post-transcriptional level [5]. MiRNAs are differentially expressed throughout embryonic development and a significant portion of these are regulators of chicken skeletal muscle growth. Computer algorithms are available for the prediction of miRNA target sites and this has become a powerful tool for understanding the signaling systems and transcriptional networks that regulate cell differentiation [1]. Studies have been reported on miRNA sequence analysis, determining homology to other species and location of genomic loci through next generation sequencing technologies [6]. Additionally, understanding miRNA expression profiles has contributed to the understanding of several biological processes including tissue development and maintenance, in which cell proliferation, cell differentiation, and the balance between the two play principal roles. These molecules have been reported to play an important role in the regulation of several processes during muscular development [7]. A combination of high sequencing and bioinformatics analysis provides a good opportunity to predict numerous novel miRNAs [8]. Identifying targets of each miRNA is crucial for understanding the biological functions of miRNAs because of the post-transcriptional nature of their regulatory effects [9, 10]. We analyzed developing chicken leg muscles at E11, E16 and P1.

Our aim was characterization of miRNA with their target mRNA, using RNA sequence to sample the transcriptome during two embryonic and one post hatch chick muscle development. Furthermore, discover miRNA patterns and group them according to biological process, molecular function and cellular component using gene ontology (GO) analysis. We hoped to gain a better understanding of the underlying functions of differentially expressed miRNAs with their target genes in chicken muscle development.

## RESULTS

### Analysis of small RNAs

**Figure 1:**
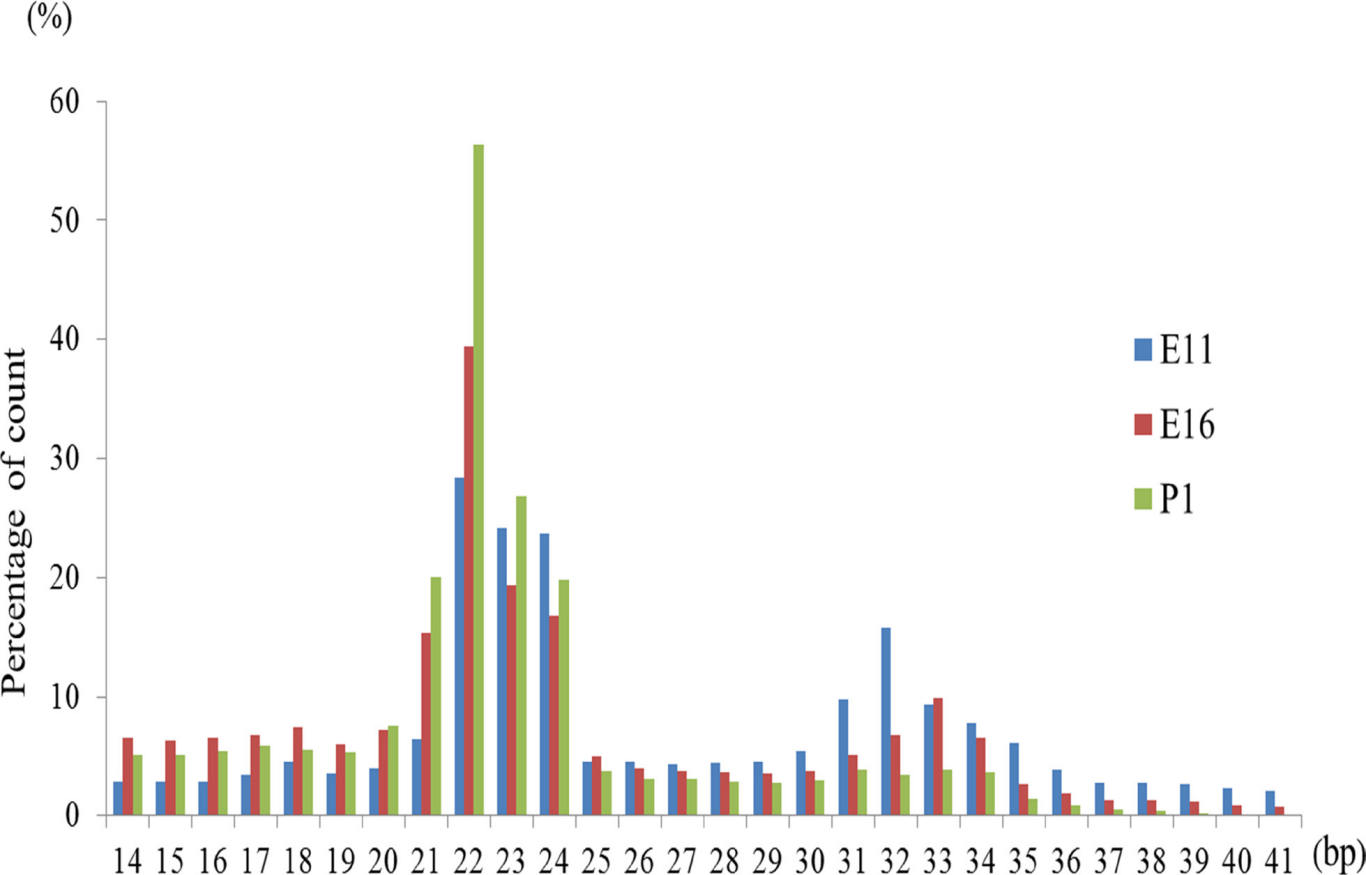
Length distribution of small RNA sequence in chicken embryonic day of 11, 16 and post hatch one day old chick (P1) The horizontal and vertical axis indicates miRNAs nucleotides and count percentage, respectively.

We established six small RNA libraries from group E11 (E11.1-E11.2), group E16 (E16.1-E16.2) and group P1 (P1.1–P1.2) yielding 6.2 to 8.9 M raw reads per library. After eliminating adaptor and low-quality reads, we obtained 4.2 to 7.4 M clean reads for these groups (Table 1). All clean reads were aligned to the chicken genome databases, miRBase, Rfam, RepBase and mRNA (Table 1). The sequence length distribution in the six libraries showed wide variation ranging from 14 to 40 nt. Most of the small RNAs were 21–24 nt in length, and 22 nt predominated as expected (Figure 1).

**Table 1:** Small RNA reads from three stages of chicken embryonic development

Samples	Total Read	Clean Read	Genome		mRNA		Mature miRBase		Rfam		RepBase	
			Aligned	Align %	Aligned	Align %	Aligned	Align %	Aligned	Align %	Aligned	Align %
E11.1	6234650	5302700	4714228	0.889	760139	0.1433	251039	0.0473	2544533	47.99%	371370	7.00%
E11.2	7925493	6556747	5728266	0.8736	1098490	0.1675	475303	0.0725	2103428	32.08%	492592	7.51%
E16.1	6512809	5359793	4816010	0.8985	693860	0.1295	965511	0.1801	1307022	24.39%	273784	5.11%
E16.2	6759531	4213112	3951219	0.9378	976328	0.2317	783613	0.186	745809	17.70%	439813	10.44%
P1.1	8992944	7112885	6611002	0.9294	805593	0.1133	2145731	0.3017	1011044	14.21%	386789	5.44%
P1.2	8872577	7469939	6983250	0.9348	1330233	0.1781	1836149	0.2458	848175	11.35%	421541	5.64%

### Identification of known and novel miRNA

**Figure 2:**
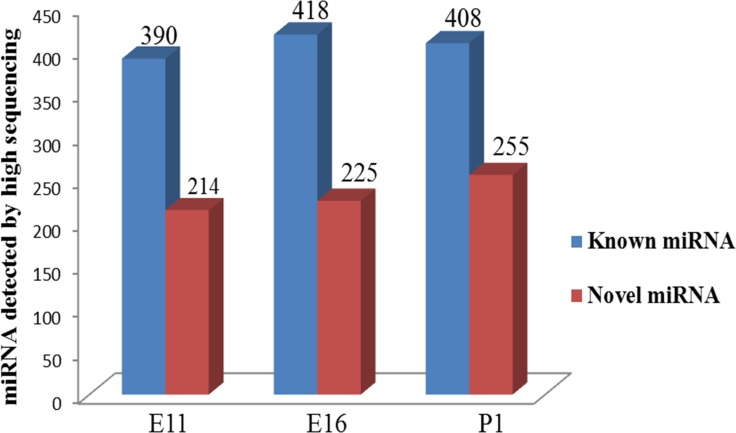
Statistics of the number of known (Blue) and novel (Red) miRNA detected by deep sequencing in chicken embryonic day of E11, E16 and post hatch one day old chick (P1)

We analyzed the sequence data for the miRNAs 390, 418 and 375 known miRNAs were detected at embryonic at E11, E16, and P1 (Figure 2). The 20 most abundant miRNAs in the three groups were ordered by the average proportion of each miRNA and included miR-148a-3p, miR-22-3p, miR-10b-5p, miR-181a-5p, miR-133a-3p, miR-126-5p, let-7f-5p, miR-10a-5p, miR-30c-5p, miR-146c-5p (Supplementary Table 1). In this group, miR-10b-5p, miR-148a-3p and miR-133a-3p were the most abundant accounting for 79,359, 171,817 and 404,782 of total normalized miRNA reads from the E11, E16 and P1 libraries, respectively.

The muscle specific miRNAs (myo-miR), miR-133a/b, miR-206, miR-486, miR-26a, miR-27b, miR-378, miR-148a and miR-181 were highly enriched in skeletal muscle and play key roles in skeletal muscle metabolism [11]. Four myo-miRs, miR-1, miR-133a, miR-133b, and miR-206, together account for nearly 25% of miRNA expression in skeletal muscles in both humans and mice [12]. Another important feature of deep sequencing is also the ability to detect novel miRNAs from Srna transcriptome. We used the miRDeep2 algorithm to identify novel miRNAs and we also identified 214, 225, and 255 potential novel miRNAs from the E11, E16 and P1 libraries, respectively (Figure 2). These putative novel microRNAs constituted a larger fraction of the total sequenced reads in the P1 sample compared with the E11 and E16 samples (Figure 2). However, the expression levels of the novel miRNAs were relatively lower than the known miRNAs. We found 35 novel miRNAs in common between E11, E16 and P1 with count reads were greater than 1000. These included 7_32745, 13_9282, 17_11642, 7_32911, 4_27031, 15_10888, JH375593, 1_38571, 27_20584, 24_19516 and 4_25848. In addition, 29 of these novel miRNAs shared homology with known miRNAs in their seed sequence and indicated that they may be new family members of those known miRNAs. The novel miRNAs 7_32745, 17_11642, 7_32911 and 15_10888 were homologous with miR-10a-5p, miR-181a-5p, miR-26-5p and miR-130a-3p respectively (Supplementary Table 2).

### Identifying miRNAs expression and differentially expressed miRNAs

We used RNA sequencing to identify differentially expressed miRNAs in our miRNA libraries.

**Figure 3:**
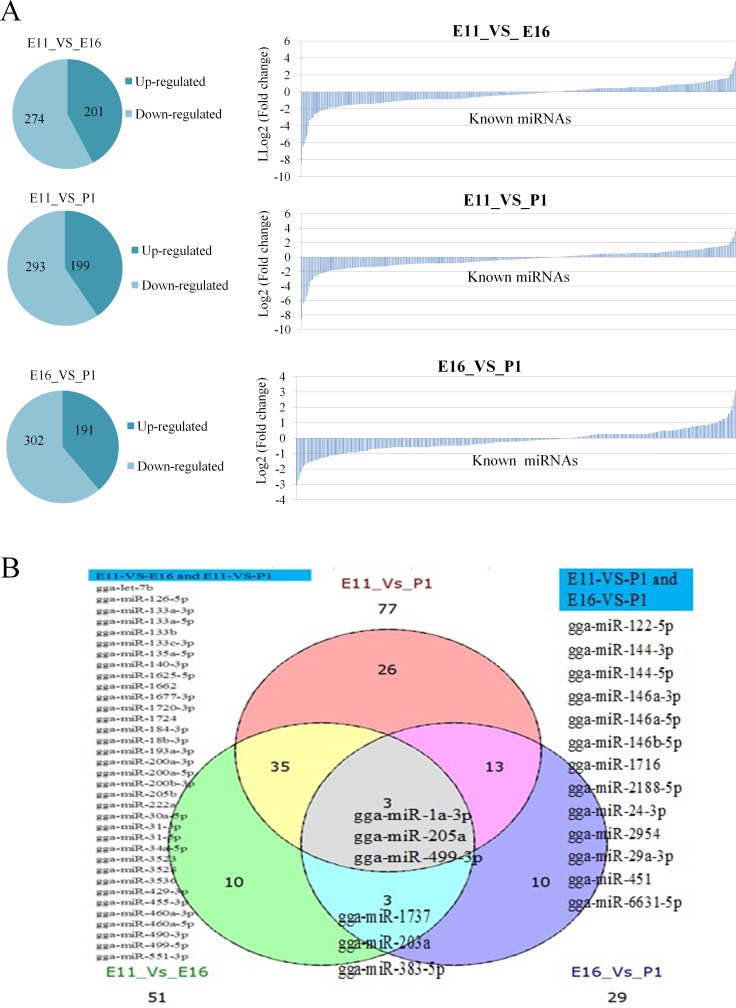
Differentially expressed known miRNAs in each contrast group (**A**) Differentially expressed up and down regulated miRNAs, and log2 fold change (vertical axis) and predicted miRNAs (horizontal axis). (**B**) Differentially expressed miRNAs with *P* < 0.05.

Pairwise comparisons indicated that 475 (201 up regulated, 274 down regulated), 492 (199 up regulated, 293 down regulated), 493 (192 up regulated, 302 down regulated) miRNAs were identified, in E11_VS_E16, E11_VS_P1 and E16_VS_P1, respectively (Figure 3A). Some miRNAs such as miR-203 had been previously reported as up regulated in proliferating myoblasts and down regulated during differentiation at days E14 and E16 [13]. We found 51, 77 and 29 miRNAs that were differentially expressed |log2fold change| > 1 and (*P* < 0.05) in E11_VS_E16, E11_VS_P1 and E16_VS_P1, respectively. In these three comparison groups there were at least three miRNAs (miR-205a, miR-1a-3p and miR-499-3p) that were shared equally (Figure 3B).

### Identifying mRNAs expression and differentially expressed mRNAs

**Figure 4:**
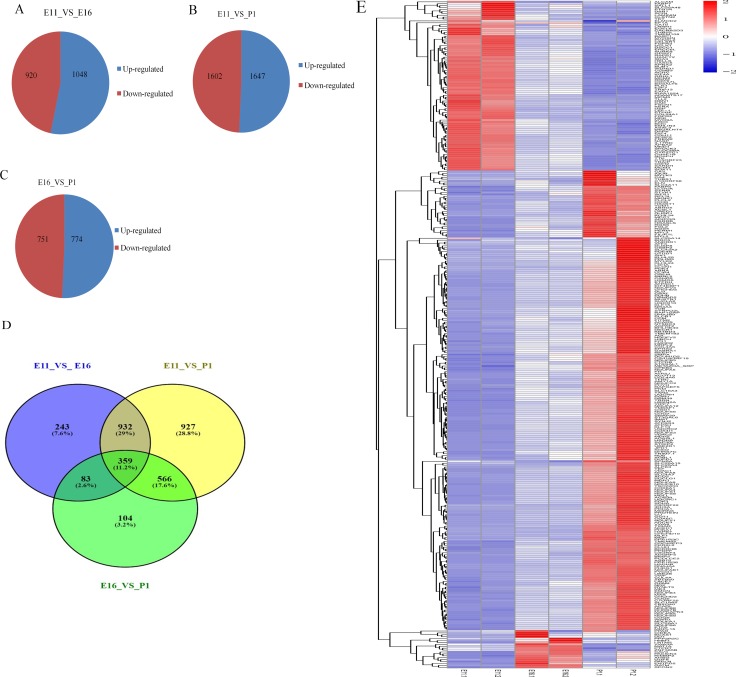
Differentially expressed mRNAs in each contrast group (**A**–**C**) Differentially expressed mRNA up and down regulated with absolute value (log2 fold change ≥ 1) in three contrast group. (**D**) Venn diagram shows differentially expressed mRNA with *P* < 0.0059. (**E**) Heat map of common up and down regulated differentially expressed mRNA in three contrast group. Interaction networks of differentially expressed miRNAs and genes in each contrast group.

We performed RNA sequencing to identify genes that were differentially expressed at our three sampling times. We then identified mRNA expression 1,968 (1,048 up regulated, 920 down regulated), 3,249 (1,647 up regulated, 1,602 down regulated) and 1,525 (774 up regulated, 751 down regulated) in E11_VS_E16, E11_VS_P1 and E16_VS_P1 respectively (Figure 4A–4C). In these groups of differentially expressed genes, 1,636, 2,819 and 1,117 showed significant changes (|log2fold change| ≥ 1 and (*P* < 0.05) in mRNA levels in E11_VS_E16, E11_VS_P1 and E16_VS_P1, respectively. We also found that 359 mRNAs were shared equally between the three comparison groups. (Figure 4D). Furthermore, those mRNAs in common were equally shared across the three groups, and java tree views were used to show the heat map of the differentially significant expressed mRNA (Figure 4E).

### Integrated analysis of differentially expressed genes and miRNAs

**Figure 5:**
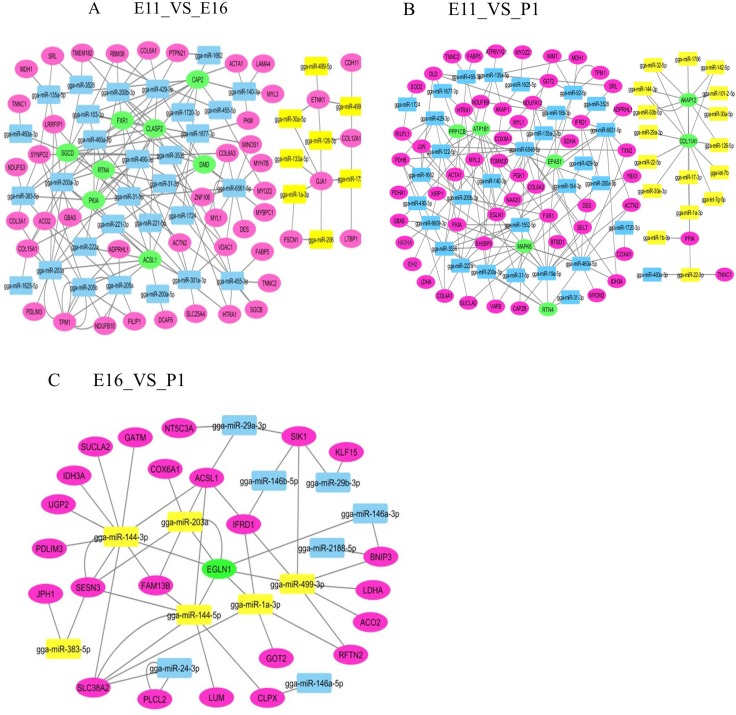
Interaction networks of differentially expressed miRNAs and genes in each contrast group (**A**) E11_VS_E16 (**B**) E11_VS_P1 (**C**) E16_VS_P1. Green ellipse; interaction with more than six miRNAs. Purple ellipse; interaction with less than five miRNAs. Yellow square; up regulated miRNA. Blue square; down regulated miRNA.

We further compared the mRNA transcriptome with the miRNA samples to identify potential links between miRNAs and mRNA expression. Target genes of the miRNAs were predicted on the basis of chicken sequences using the target scan (http://www.targetscan.org) [14, 15]. The integrated analysis of mRNA and miRNA interactions indicated an overall negative correlation or regulation with absolute value of log2 (fold change) ≥ 1.75, *P* < 0.005. We identified 288 miRNA-gene pairs (189 negative and 99 positive) for E11_VS_E16, 441 pairs (188 negative and 253 positive) for E11_VS_P1 and 150 pairs (53 negative and 97 positive pairs) for E16_VS_P1. The negative and positive correlations of miRNA and mRNA were normalized based on the miRNA-target gene mean values of > 20 for E11_VS_E16 and E16_VS_P1 and > 30 for E11_VS_P1 (Figure 5).

We performed an integrated analysis according to the negative correlation of differentially expressed genes and the miRNAs between the three comparison groups. The networking interactions were drawn using Cytoscape 3.0.(http://cytoscape.org/). A negative regulation model was expected since miRNAs can directly initiate mRNA degradation or inhibit mRNA translation [16]. To better understand the interaction between differentially expressed miRNA-genes network was constructed in three comparable groups (Figure 5). In the E11_VS_E16 contrast group, the network consisted of 53 nodes for mRNA, 38 nodes for miRNA and 174 edges that showed 174 regulatory events between target mRNA and miRNAs (Figure 5A). Furthermore, in the E11_VS_E16 contrast group, 8 miRNAs (miR-203a, miR-6561-5p, miR-460a-5p, miR-205a, miR-3536, miR-103-3p miR-205b, and miR-200b-3p) were highly regulated. In this group, miR-6561-5p, miR-203a, miR-3536 and miR-460a-5p potentially targeted 12, 10, 9 and 8 mRNAs, respectively. This indicated that these miRNAs most likely significantly contributed to the regulation of mRNA expression during chicken embryo skeletal muscle development in the E11_VS_E16 contrast group (Figure 5A). Networking interactions showed that miR-460a-5p targeted the eight mRNAs *ACTN2, CAP2, SGCD, RTN4, PIKA, COL15A1, FXR1 and CLASP2*.

In the E11_VS_P1contrast group, the network consisted of 61 nodes for mRNA, 48 nodes for miRNA and 188 edges that showed 188 regulatory events between target mRNA and miRNAs (Figure 5B). Moreover, in the E11_VS_P1 contrast group, eleven miRNAs (miR-6548-5p, miR-19a-5p, miR-3536, miR-6631-5p, miR-222a, miR-140-3p, miR-92-5p, miR-135a-5p, miR-455-3p, miR-460a-5p and miR-200a-3p), were highly regulated. Of these miRNAs, miR-6548-5p, miR-6631-5p, miR-92-5p and miR-140-3p potentially regulated of 15, 9, 7 and 7 mRNA respectively. The networking interactions in these contrast groups identified miR-19a-5p as targeting *XIRP*1, *YBX*3, *ATP*1B1, *MAPK*6, *EGLN*1, *MAPK*6, *CAPZB*, *SELT*, *EPAS*1, *NDUFA*12, *ACTN*2, *RTN*4 and *BTBD*1.

In E16_VS_P1contrast group, consists of 24 nodes for mRNAs, 13 nodes for miRNAs, and 53 edges that show 53 regulations between target mRNA and miRNAs (Figure 5C). In this contrast group, we could identify the target genes of five miRNAs (miR-499-3p, miR-144-3p, miR-144-5p, miR-203a and miR-1a-3p) (Figure 5C). Among these miRNAs, miR-499-3p, miR-144-3p and miR-1a-3p, potentially targeted 6, 6 and 5 mRNAs respectively. The miR-499-3p potentially targeted, six mRNAs including (*EGLN1, BNIP3, RFTN2, LDHA, ACD2 and SIK1*). Since a single miRNA can potentially regulate multiple genes, the mRNA expression profiles are also dependent on the miRNA expression level. Therefore, the integrative analysis allowed us to identify miRNA-mRNA interaction networks potentially involved during E11, E16 and P1 embryonic stages. We have identified a number of miRNAs with predicted target genes associated with chicken embryonic skeletal muscle development, and thus miRNA may decrease gene expression level.

### qPCR validation of sequencing data

**Figure 6:**
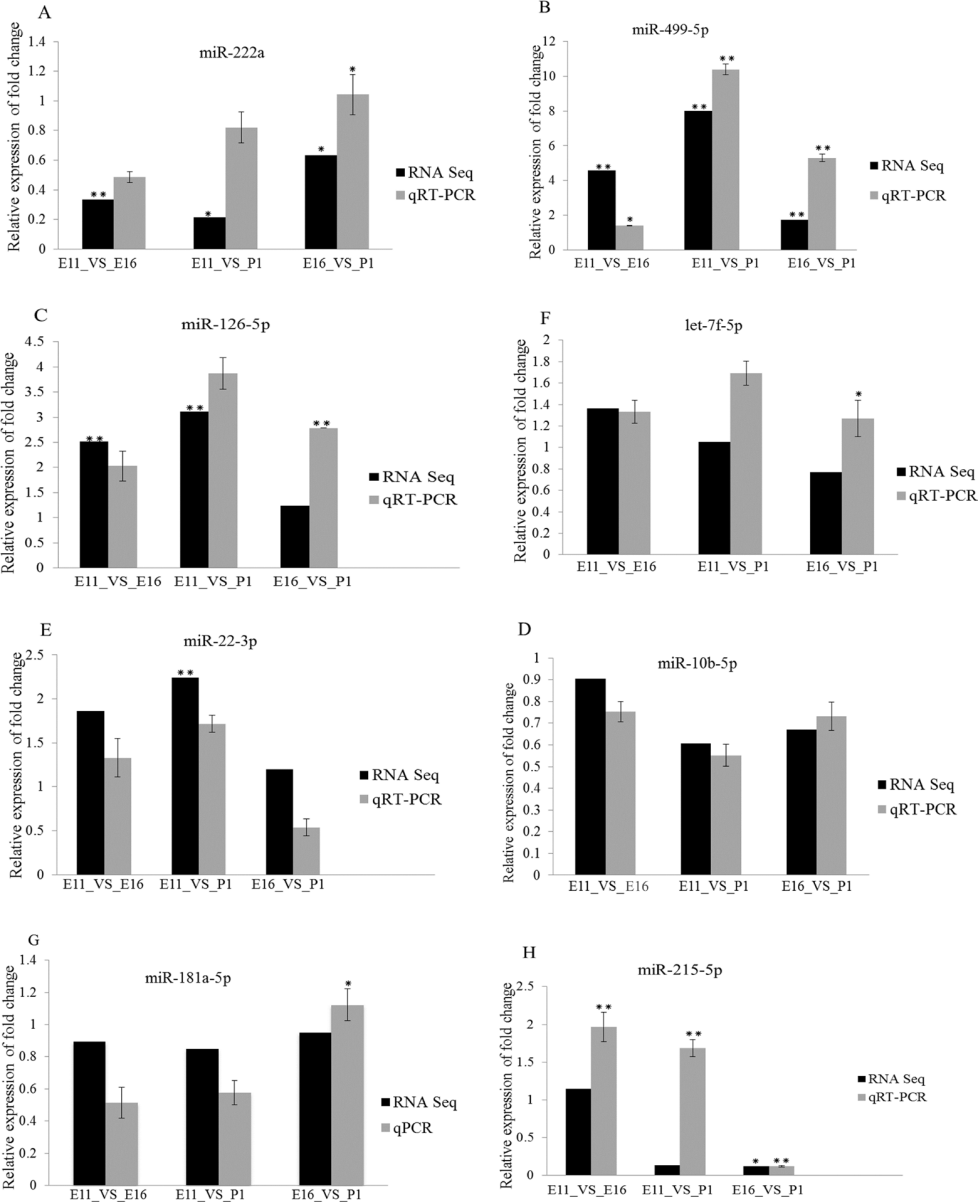
RT-qPCR validation of eight differentially expressed miRNAs in three comparisons, E11_VS_E16, E11_VS_P1 and E16_VS_P1 (**A**–**H**) The vertical axis is represented relative fold change expression of miRNAs and the horizontal axis indicated contrast groups. The numerical data are shown as means ± S.E.M. of at least three replicates. The asterisks denotes ^**^P < 0.01 and ^*^P < 0.05.

**Figure 7:**
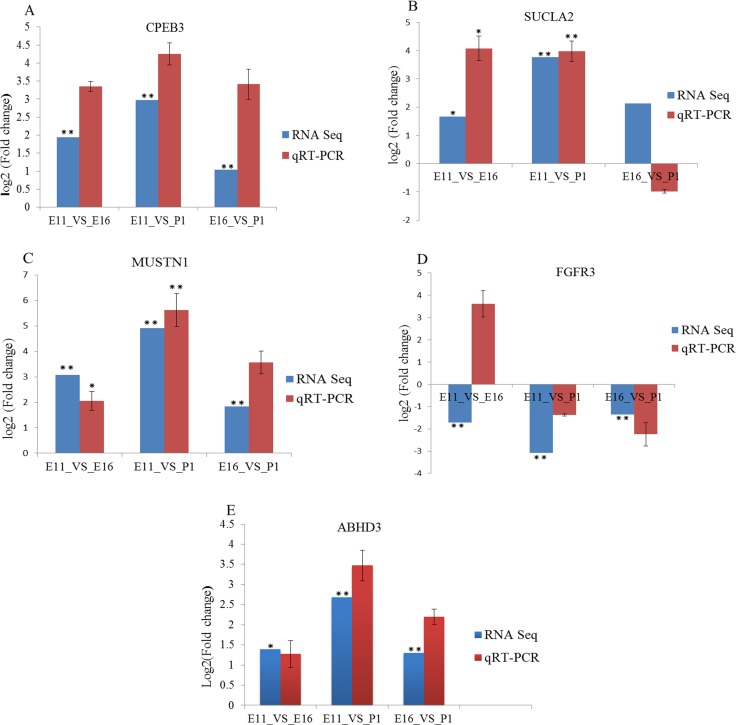
RT-qPCR validation of five differentially expressed mRNA in three contrast groups (**A**–**F**) The expression levels of genes, (*CPEB3, SUCLA, MUSTN1, FGFR3* and *ABHD3*) were normalized against β-actin gene. The vertical axis represented Log2 (Fold change) of genes and the horizontal axis indicated contrast groups. The numerical data are shown as means ± S.E.M. of at least three replicates. The asterisks denotes ^*^P < 0.05 and ^**^P < 0.01.

To validate the miRNAs and mRNA expression, determined by sequencing data, we selected eight miRNA and five mRNA based on their expression profile. These included miRNAs (miR-222a, miR-499-5p, miR-126-5p, miR-10b-5p, miR-22-3p, let-7f-5p, miR-181a-5p and miR-215-5p) (Figure 6) and differentially expressed genes (*CPEB3, SUCLA2, MUSTN1, FGFR3* and *ABHD3*) (Figure 7). The validation and sequencing result were showed similar patterns of expression in the three contrast group (Figures 6 and 7). Thus, deep sequencing and in silico analysis were reliable techniques for establishing differentially expressed miRNAs and mRNAs in the chicken embryonic muscle samples.

### Target genes prediction and GO analysis

**Figure 8:**
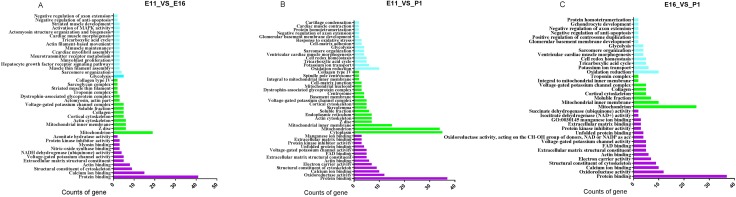
GO enrichment of the up-and down-regulated differentially expressed genes |Log2Fold change > 1.75| with *P*-values < 0.001 (**A**–**C**) Biological process, cellular component and molecular function. The Y axis is indicated GO term and X axis is number of genes.

We used differentially expressed mRNAs (*P* < 0.05) were that negatively correlated with miRNA levels for gene ontology term and Kyoto Encyclopedia of Genes and Genomes (KEGG) pathway analysis for each comparison. GO term enrichment analysis detected significantly over represented GO terms in differential expressed gene with *P-value* < 0.05. The most significantly enriched GO terms in biological processes, cellular components and molecular functions for E11_VS_E16, E16_VS_P1 and E11_VS_P1 comparison groups (Figure 8). GO is gene functional classification system which offers a dynamic-updated vocabulary to widely describe properties of genes and their products in an organism [17, 18]. We performed GO analysis by using differential expressed gene against the GO (http://www.geneontology.org/). We analyzed relevant information for these three GO groups that were associated with chicken embryo skeletal muscle development. The biological processes included genes required for muscle maintenance, myoblast proliferation, muscle thin filaments, MAPK activity and cardiac myofibril assembly. The classification of molecular function identified structural cytoskeleton constituents, NADH dehydrogenase (ubiquinone) activity, striated muscle development, myosin binding, protein kinase inhibitor activity and hexokinase activity in the E11_VS_E16 comparison (Figure 8A). In the cellular components category, we found significant differentially expressed genes related to the soluble fraction, mitochondrial inner membrane, collagen, actin cytoskeleton.

In the E11_VS_P1 comparison, the significant differentially expressed genes included negative regulation of axon extension, sarcomere organization, and cell redox homeostasis and kinase inhibitor activity. In the molecular function category, extracellular matrix and cytoskeleton structural elements were identified. The cellular component category included mitochondrial inner membrane, mitochondrion, cell-matrix junction and spindle pole centrosome (Figure 8B).

In the E16_VS_P1 comparison, the differentially expressed genes were involved in oxidation reduction, negative regulation of anti-apoptosis, negative regulation of axon extension, potassium ion transport identified in biological process. While molecular function of GO term which related with muscle development, identified based on extracted differential expressed gene targeted of miRNA are protein binding, protein kinase inhibitor activities, succinate dehydrogenase (ubiquinone) activity, and actin binding (Figure 8C).

### KEGG pathway enrichment analysis

KEGG is a database collection of gene products related to metabolism and other cellular processes [19]. We identified significantly enriched biological pathways related to the differentially expressed genes using KEGG pathway analysis. In the E11_VS_E16 comparison group, we identified 23 pathways and eight pathways were significantly enriched. Of those eight, the highest level of significance was reached for genes involved in gluconeogenesis, calcium signaling, glyoxylate and dicarboxylate metabolism, oxidative phosphorylation, the TCA cycle and pyruvate metabolism (Table 2 and Supplementary Table 3). In the E11_VS_P1 comparison group, 36 pathways were assigned to differentially expressed genes and 14 of those were significantly enriched. The highest levels of significance were the TCA cycle, pyruvate, propanoate and butanoate metabolism, oxidative phosphorylation and Wnt signaling (Table 2 and Supplementary Table 3).The Wnt signaling pathway regulates the expression of myogenic regulatory factors that are vital for myogenic ancestry development and the formation of functional multinucleated myotubs [20], [21]. Also the other biological pathway, such as cytokine-cytokine receptor interaction, Wnt signaling pathway, regulation of actin cytoskeleton, tight junction, and cell cycle which have been involved in cell and tissue structure [22].

**Table 2:** KEGG pathway analysis of differentially expressed up and down regulated gene (fold change > 1.75) in three contrast groups, (E11_VS_E16, E11_VS_P1, E16_VS_P1)

Contrast group	Pathway	Count	*P*-value	*q*-value
E11_VS_E16	Glycolysis/Gluconeogenesis	3	3.28E-05	5.97E-06
	Calcium signaling pathway	4	4.79E-05	7.98E-06
	Glyoxylate and dicarboxylate metabolism	2	1.10E-04	1.63E-05
	Reductive carboxylate cycle (CO2 fixation)	2	1.10E-04	1.63E-05
	Oxidative phosphorylation	3	5.09E-04	5.66E-05
	Citrate cycle (TCA cycle)	2	6.43E-04	6.76E-05
	Pyruvate metabolism	2	9.75E-04	9.75E-05
	Cysteine metabolism	1	0.011454	9.54E-04
E11_VS_P1	Citrate cycle (TCA cycle)	6	3.65E-12	2.44E-12
	Pyruvate metabolism	5	2.10E-09	8.41E-10
	Glycolysis / Gluconeogenesis	5	9.91E-09	3.30E-09
	Oxidative phosphorylation	5	1.06E-06	2.12E-07
	Butanoate metabolism	3	1.12E-05	1.67E-06
	Wnt signaling pathway	4	5.15E-05	5.72E-06
	Valine, leucine and isoleucine biosynthesis	2	5.77E-05	6.07E-06
	Cysteine metabolism	2	7.68E-05	7.31E-06
	Propanoate metabolism	2	7.44E-04	5.51E-05
	Calcium signaling pathway	3	0.001658	1.08E-04
	MAPK signaling pathway	3	0.004502	2.43E-04
	Apoptosis 2	2	0.005367	2.75E-04
E16_VS_P1	Citrate cycle (TCA cycle)	5	7.51E-12	6.14E-12
	Glycolysis / Gluconeogenesis	4	2.40E-08	1.20E-08
	Pyruvate metabolism	3	1.57E-06	4.49E-07
	Cysteine metabolism	2	1.38E-05	2.75E-06
	Oxidative phosphorylation	3	6.24E-05	1.04E-05
	Arginine and proline metabolism	2	1.71E-04	2.44E-05
	Glycine, serine and threonine metabolism	2	2.89E-04	3.85E-05
	Alkaloid biosynthesis I ___	1	0.004309	4.42E-04
	Nucleotide sugars metabolism	1	0.004309	4.42E-04
	Valine, leucine and isoleucine biosynthesis	1	0.005025	4.79E-04
	Phenylalanine, tyrosine and tryptophan biosynthesis	1	0.006457	5.61E-04
	Glyoxylate and dicarboxylate metabolism	1	0.007886	6.31E-04
	Reductive carboxylate cycle (CO2 fixation)	1	0.007886	6.31E-04
	Pentose and glucuronate inter conversions	1	0.0086	6.37E-04

In the E16_VS_P1 comparison, we identified 30 pathways assigned for differentially expressed genes and 13 were significantly enriched. The most significant were genes involved in cysteine, arginine and proline metabolism, oxidative phosphorylation, glycolysis and the TCA cycle (Table 2 and Supplementary Table 3). Previous reports of miRNA regulation of myogenesis have found single genes [23, 24]. However, growing evidence suggests that miRNA can also have an effect on signal transduction pathways [25].

### Verification of the interaction between miRNA and target gene

**Figure 9:**
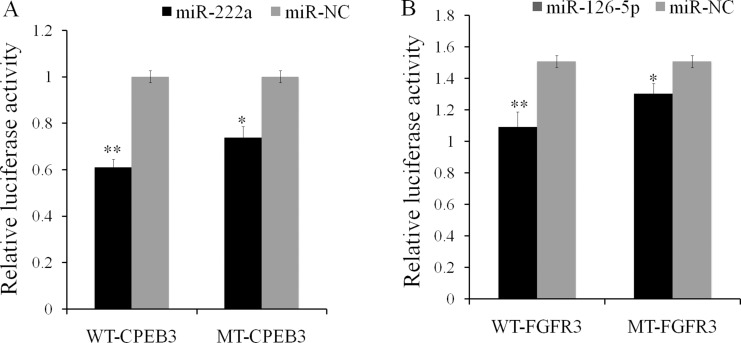
Validation of the miR-222a and miR-126–5p predicted targets using luciferase reporters containing CPEB3 and FGFR3 3′UTRs in DF-1 cells (**A**) *CPEB3* target regulated by gga-miR-222a (**B**) *FGFR3* target down regulated by gga-miR-126–5P. The luciferase activity of the transfected with the PGLO vector containing the *CPEB3* and *FGFR3* 3’UTR fragment with the binding sequence of miR-222a and miR-126–5p was inhibited by transfection of miR-222a and miR-126–5p mimic into DF-1 cells line respectively. The Y axis represented the relative fold change of Renilla luciferase activities compared to the level of the negative control. The numerical data are shown as means ± S.E.M. of at least three replicates. The asterisks denotes ^*^P < 0.05, ^**^P < 0.01.

**Figure 10:**
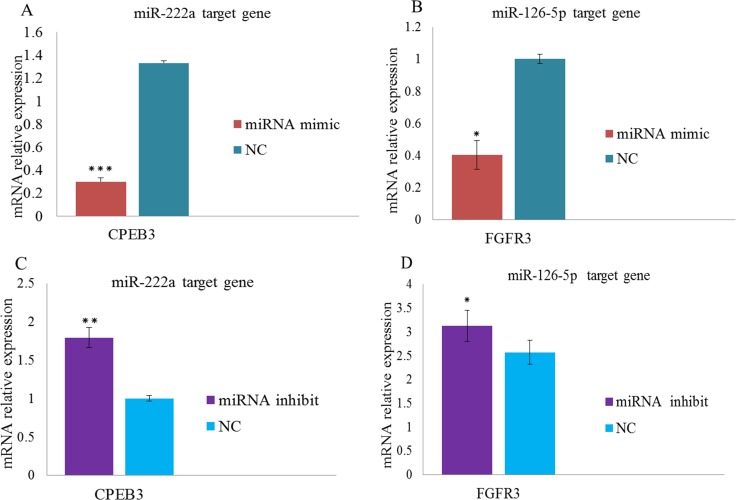
Overexpression of miRNAs inhibits mRNA expression levels in DF-1 cell lines (**A**–**B**) Over expression of miR-222a and miR-126–5p causes decreases *CPEB3* and *FGFR3* target gene expression levels respectively. (**C**–**D**) miR-222a and miR-126–5p inhibition causes an increase the expression levels of *CPEB3* and *FGFR3* target genes respectively. Mimics and control oligonucleotides were transfected into DF-1 cells for 48 h to detect target gene expression levels. The fold change values were normalized using the comparative 2^-∆∆CT^ method [∆∆CT= ∆CT _(target gene)_ -∆CT _(reference gene)_] from at least three independent experiment. The numerical data are shown as means ± S.E.M. of at least three replicates. The *P*-values (^*^P < 0.05, ^**^P < 0.01 *and* ^***^P < 0.001*)* compared with the NC groups.

We constructed dual luciferase reporters to test whether miR-126-5p and miR-222a bind directly to the 3′-untranslated regions (3′-UTR) of *CPEB3* and *FGFR3* mRNAs, respectively. Both miR-222a and miR-126-5p significantly reduced the firefly luciferase activity of the wild type and mutants of the *CPEB3* and *FGFR3* reporters compared with negative control respectively (Figure 9A and 9B).We also evaluated the effects of miR-222a and miR-126-5p by transfecting miR-222a and miR-126-5p mimics or inhibitors separately in DF-1 cells. We then measured target gene expression after 48 h. Both miR-222a and miR-126-5p down regulated their respective targets (Figure 10A and 10B). When miR-222a and miR-126-5p inhibitors were transfected, *CPEB3* and *FGFR3* increased respectively (Figure 10C and 10D). This correlation analyses provided support for the results using genome and in silico analysis.

## DISCUSSION

In the present study, we identified miRNAs by deep sequencing from three embryonic stages in Xinghua chickens. These libraries represented 390, 418 and 375 known miRNAs and 214, 225 and 255 novel miRNAs in chicken skeletal muscle development. Among the known miRNAs, miR-10b-5p, gga-miR-148a-3p, gga-miR-22-3p and gga-miR-133a-3p were relatively high expressed in all three stages of chicken skeletal muscle development. Four myo-miRs (miR-1, miR-133a, miR-133b, and miR-206) accounted for nearly 25% of miRNA expression in skeletal muscle in both humans and mice [12]. The chicken embryo has been an especially useful vertebrate system for developmental biologists owing to experimental advantages of *in vivo* embryogenesis [24].

In these analyses, predicted ncRNA (noncoding RNA) pseudogenes were greatly reduced relative to the numbers found in their human ncRNA counterparts. The chicken ncRNA predictions therefore represent a functional set. If ncRNA genes maintain their placement with respect to neighboring genes, chicken ncRNA gene locations could be used to identify which mammalian copies are likely to be functional and which are probable pseudogenes [27]. We analyzed differentially expressed miRNAs among the three contrast groups. We found fewer miRNAs in the E11_VS_E16 comparison than in the E11_VS_P1 and E16_VS_P1 comparisons. This indicated that there are large differences in P1 compared with the E11 and E16 groups. The differentially expressed mRNAs in the E11_VS_P1 comparison were greater than either the E11_VS_E16 or the E16_VS_P1 comparisons. MiRNAs play a vital role in a number of cellular and biological processes such as cellular differentiation [26]. Regulation of miRNA expression has been associated with several pathologies [27]. MiRNA and mRNA expression studies provide important information that can be further validated.

MiR-221 and miR-222 have been found to be modulated during myogenesis and to play a role both in the progression from myoblasts to myocytes and in the achievement of the fully differentiated phenotype [28]. *CPEB3* expression was significantly and negatively correlated with miR-222a levels and *FGFR3* was negatively correlated with miR-126-5p indicating that these genes are targets for these miRNAs. The *CPEB3* and *FGFR3* 3′-UTR sequences around the miRNA-222a and miR-126-5p target sites and the seed sequence of mature miRNA-222a and miR-126-5p are well conserved in chickens. In the current study, we also performed *in vitro* reporter gene assays for target gene validation using the DF-1 cell line. The fibroblast growth factors receptor (*FGFR*) has been shown to be intimately involved in fetal skeletal muscle growth and development of cultured skeletal muscle *in vitro* [29]. *CPEB3* is the remarkable conservation of the genomic that was a good enough indicator of the existence of a novel alternative 3’-UTR isoform that have been confirmed by bioinformatics analyses [30]. We observed in this study that several miRNA families were negatively correlated with mRNA expression levels. Previous studies have indicated that clustered miRNAs are processed from the same primary transcript [31] and that intronic miRNAs are from the same primary transcript as their host gene [32]. Recognizing mRNAs regulated by miRNAs will help us better understand the biological functions of miRNAs [33]. We constructed interaction networks of the differentially expressed miRNAs and mRNA for the three contrast groups. In the E11_VS_E16 group, miR-6561-5p, miR-203a, miR-3536 and miR-460a-5p were predicted to regulate their cognate target genes. In E11_VS_P1, miR-6548-5p, miR-6631-5p, miR-92-5p and miR-140-3p were regulators. In the E16_VS_P1 group, miR-499-3p, miR-144-3p and miR-1a-3p were target gene regulators.

The RNA Seq data also helped us to investigate biological functions in muscle development in chicken embryos. The mRNA-miRNA pairs were classified into functional categories involving biological processes. The expressed genes that were most represented were those involved in negative regulation of axon extension (*RTN4*), the TCA cycle (*ACO2*, *MDH1*, *SDHA*, *IDH3A*) glycolysis (*PGK*, *Q5F426_CHICK, LDHA, MDH1, PGAM1, HK1*) and sarcomere organization (*ACTB*). Although we found some differences between the three contrast groups, the primary terms and general biological functions were similar. However, myoblast proliferation (*CACNA2D1*), muscle thin filament assembly (*ACTA1*), activation of MAPK activity (*CACNA2D1*), muscle maintenance (*DMD*), and negative regulation of anti-apoptosis (*RTN4*) were major terms for the E11_VS_E16 group. The E11_VS_P1 group had the terms cartilage condensation (*COL11A1*), protein homo-tetramerization (*SOD2, ACTN2*) and cell-matrix adhesion (*CTNNB1, NID1*) as the major terms. The E16_VS_P1 contrast group main terms included glomerular basement membrane development (*NID1*), cell redox homeostasis (AIFM1, SELT, TXN2, DLD) and oxidation reduction (*AIFM1, EGLN1, DLD, SDHA, HADHA, IDH3A, SOD2, PDHA1*).

Apoptosis is critically important for the survival of multicellular organisms by eliminating damaged or infected cells that may interfere with normal function [34]. In mammals, the Akt and the Mapk/Erk1/2 pathways are signaling pathways related to exercise [35]. Although the principal action of the Akt pathway is the metabolic regulation by insulin and insulin growth factors, the Mapk/Erk1/2 pathways are responsible for proliferation and differentiation. This pathway is also activated by other growth factors and mitogens and can be nutritionally regulated [36].

KEGG pathway analyses of targets based on the prediction data suggested that these targets were significantly enriched for oxidative phosphorylation, pyruvate metabolism, phenylalanine, tyrosine and tryptophan biosynthesis and Wntsignaling. During adult skeletal muscle regeneration, Wnt signaling is involved in satellite cell proliferation and differentiation as well as self-renewal [37]. The Wnt signaling pathway also simultaneously promotes myogenic and inhibits adipogenic differentiation within primary adult myoblasts [38].

## MATERIALS AND METHODS

### Ethics statement

All animal experiments were handled in compliance with and approved by the Animal Care Committee of South China Agricultural University (Guangzhou, People’s Republic of China). All efforts were made to minimize suffering to animals.

### Chicken embryo incubation and tissue collection

On this study we used fertilized eggs of native Chinese yellow meat type chicken (known as Xinghua chicken breeds). Eggs were incubated at 37.5°C and 78% relative humidity. Skeletal leg muscle were used at 11 day embryo, 16 day embryo and post hatch one day old chick. All fresh leg muscle tissue samples were collected, frozen in liquid nitrogen, and stored at −80°C until RNA extraction. Each stage was selected according to specific physiological characteristics of the muscle tissue. The sex of the chicken was determined by PCR amplification using sex-specific primers. Chickens with two bands of 600 bp and 450 bp were born as females; whereas that with one band of 600bp was born as males [39].

### Small RNA library construction and sequencing

Total RNA was extracted from two embryonic stages and one post-hatch chick leg muscle tissues, using TRIzol Reagent (Invitrogen, CA, USA), following manufacturer protocol. Each stage had two samples, and the total samples were six (three group × two sample/group) used for further experiment. The RNA quality and concentration was analyzed by using gel-electrophoresis and UV spectroscopy. Total RNA from each, two embryonic stages and one post hatch of chick samples were used for RNA sequencing by the Illumina Hiseq2500 system (Supplementary Figure 1).

### Identification of differentially expressed miRNAs

Based on deep sequencing data known miRNAs were identified by reading count on skeletal muscle development from two embryonic stages and one post hatch Xinghua chicken. To compare miRNAs expression level between the E11, E16 and P1 groups, expression of each miRNA was normalized by total reads.

### Differentially expressed miRNAs and genes network construction

The differential expression of mRNA-miRNA pairs were constructed into interaction networks for the three contrast groups E11_VS_E16, E11_VS_P1 and E16_VS_P1. Network construction involved interactions between miRNAs and mRNA. The putative target genes of differentially expressed miRNAs were predicted using miRBase (http://www.mirbase.org) and Targetscan software (http://www.targetscan.org). Some studies have reported an inverse correlation between the expression patterns of miRNAs and their targets [40]. The mRNA expression patterns in opposition to their corresponding miRNA levels were selected based on mRNA value and miRNA mean values. The deferentially expressed mRNAs Seq data was normalized using a |log2fold change ≥ 1.75 for the three contrast groups as candidate targets for differentially expressed miRNAs. MiRNA-mRNA interactions were integrated to construct a possible regulatory network using Cytoscape (http://cytoscape.org/).

### Analysis of miRNA and mRNA quantification

Expressions of miRNAs were analyzed by qPCR using bulge-loop miRNA qRT-PCR primers (Ribobio, Guangzhou, China). U6 was chosen as an internal control to correct for analytical variations. Five differentially expressed genes from RNA seq data *CPEB3*, *FGFR3*, *SUCLA2*, *ABHD3* and *MUSTN1*, were selected for qPCR and analyzed and normalized with the reference gene β-actin. Real-time qPCR primers were designed using the Premier Primer 5.0 software (Premier Biosoft International, Palo Alto, CA, USA). The concentration of each primer was 20 μM. qPCR was performed on the Bio-Rad S1000 (Bio-Rad, Hercules, CA, USA) with SsoFast Eva Green Supermix (Bio-Rad) as follows: 95°C for 2 min; and 95°C for 10 s, 56°C for 30 s; and 72°C for 30 s for 40 cycles. Each reaction was performed in triplicate, and the data were analyzed by the 2^-△△Ct^ method.

### Bioinformatics analysis

Gene co-expression was determined from the differentially expressed up-regulated and down-regulated clusters. The mRNAs that were miRNAs targets were used to combine a computational prediction and experimental method based on paired miRNA and mRNA profiling. The critical miRNAs and their targets were analyzed for each contrast group. Those miRNA and mRNA pairs were used for further GO and KEGG enrichment analysis using MAS3.0 (http://bioinfo.capitalbio.com/mas3/) gene ontology database.

### Plasmid construction

The plasmid pmirGLO dual-luciferase (Promega, Madison, WI, USA)miRNA target expression vector was used for to verify miRNA regulation. The 3′-UTRs of *CPEB3* and *FGFR3* were amplified from the chicken genome and cloned into pmirGLO using the *Pmel* and *XbaI* restriction sites. The mutant *CPEB3* 3′-UTR and *FGFR3*3′-UTR plasmids were generated by changing the miR-222a and miR-126-5p binding sites from ATGTAGCA to GACGTAGT, and TAATAAT to ATCACGA, respectively. Mutagenesis was performed by PCR amplification and *DpnI* digestion.

### Cell culture and luciferase reporter assays

Cell culture and dual luciferase reporter assay using DF-1 cells were culture in Dulbecco’s modified Eagle’s medium with high glucose (Gibco, Invitrogen, Carlsbad, CA, USA) supplemented with 10% fetal bovine serum (Gibco) 1% glutamine, and 1% penicillin (Gibco). The cells were incubated at 37°C in a 5% CO2 atmosphere. Cells were transfected using Lipofectamine3000 (Invitrogen, Carlsbad, CA, USA) in 96-well plates. MicroRNA mimics for miR-126-5p and miR-222a and negative controls were synthesized by Ribobio (Guangzhou). These were co-transfected with luciferase reporters pmir-GLO wild-type and pmir-GLO mutant plasmids containing *FGFR3* and *CPEB3* 3′UTRs. Each transfection was performed in six replicates. Cells were collected and analyzed 48 h after transfection using Multi detection Microplate Reader (Biotek, Winooski, VT, USA) and a Dual-GLO Luciferase Assay System Kit (Promega). The normalized Renilla luciferase activity was compared with the control, miRNA-126-5p, miR-222a and their mutant groups using the Student’s *t*-test.

### Statistical analysis

We used Microsoft Excel for analyze statistical data. Statistical significance was determined using the Students *t*-test and *P* < 0.05 values were considered statistically significant. The numerical data were presented as mean ± standard error and the error bars in the graphs indicated the standard error of the mean (S.E.M).

## CONCLUSIONS

The work has characterized miRNA and mRNA populations from chicken skeletal muscle from two embryonic and one-post hatch chicken embryos. Differentially expressed miRNA and mRNA populations were analyzed from three contrast groups. The data identified a large number of miRNAs and their target genes of known and unknown functions. GO annotation and KEGG pathway analyses were carried on target gene that negatively correlated with miRNAs. These genes were found involving in oxidation reduction, negative regulation of anti-apoptosis, negative regulation of axon extension, kinase inhibitor activity, myoblast proliferation, muscle thin filament, MAPK activity. *In vitro* analyzed of the over expression of miR-222a and miR-126-5p was significantly reducing the expression level target gene *CBEB3* and *FGFR3* in DF-1 cells, respectively. Furthermore these result help to improved common understanding of the function miRNAs and target genes.

## SUPPLEMENTARY MATERIALS FIGURE AND TABLES






