# Supplementary material for: Characterization of miRNA and their target gene during chicken embryo skeletal muscle development
Source: Oncotarget. 2017 Nov 6;9(25):17309–24. doi: 10.18632/oncotarget.22457 (PMC5915118; doi:10.18632/oncotarget.22457)
Supplement: Supplementary file 1 [file oncotarget-09-17309-s001.pdf]

Characterization of miRNA and their target gene during chicken embryo skeletal muscle development

SUPPLEMENTARY MATERIALS

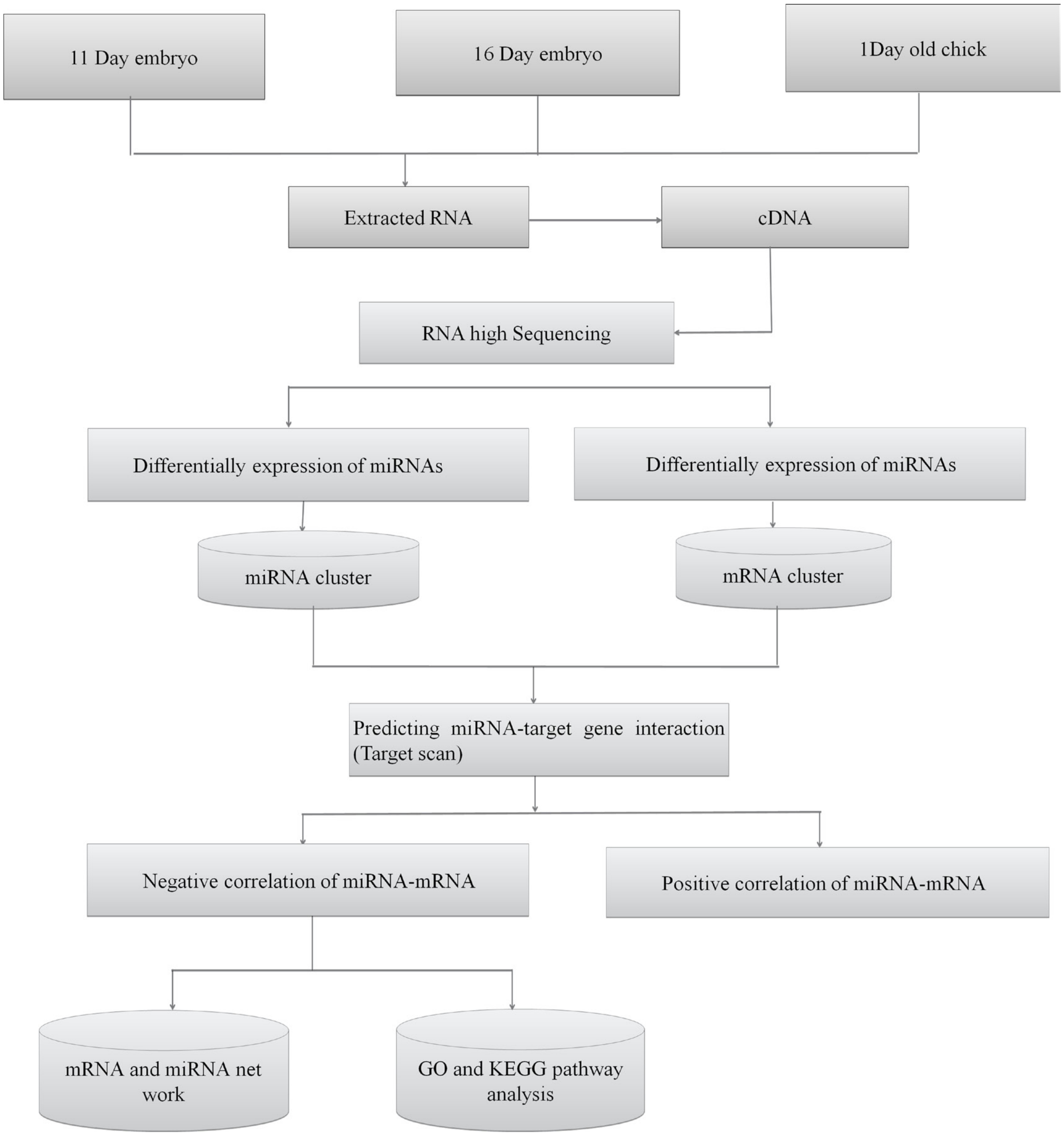

Supplementary Figure 1: General experimental work follows.

Supplementary Table 1: The most abundant miRNA in three chicken embryonic skeletal muscles development stage using deep sequencing reading count

miRNAs	E11	E16	P1
gga-miR-499–5p	4076	47917	200576
gga-miR-19b-3p	4323	6972	11312
gga-let-7c-5p	4273	9160	17417
gga-miR-128–3p	3954	9454	15694
gga-miR-222a	2387.5	1916.5	2681
gga-miR-10b-5p	79359	166001	258894
gga-miR-22–3p	29627	127427	346533
gga-miR-148a-3p	60674	171817	324459
gga-miR-181a-5p	47158	116807	246801
gga-miR-10a-5p	69134	53119	126970
gga-let-7f-5p	16049	60235	103238
gga-miR-126-5p	13034	76518	209127
gga-miR-27b-3p	4689	15322	48240
gga-miR-101–3p	6315	18858	47562
gga-miR-100–5p	21441	24521	46578
gga-miR-30c-5p	11746	44679	98333
gga-miR-146c-5p	12409	19031	33823
gga-miR-133a-3p	3562	95580	404782
gga-miR-21–5p	3748	10982	20793
gga-miR-499–5p	4076	47917	200576

E11, eleven embryo day, E16 Sixteen embryo day and P1, Post hatch one day old chick.

Supplementary Table 2: Discovered novel miRNAs and homology with known miRNA in their seed sequences. See_Supplementary_Table 2

Supplementary Table 3: KEGG pathway analysis of differentially expressed up and down regulated gene in three contrast groups, (E11_VS_E16, E11_VS_P1, E16_VS_P1). See_Supplementary_Table 3
